# Supplementary material for: Analytic optimization of Plasmodium falciparum marker gene haplotype recovery from amplicon deep sequencing of complex mixtures
Source: PLOS Glob Public Health. 2024 May 30;4(5):e0002361. doi: 10.1371/journal.pgph.0002361 (PMC11139333; doi:10.1371/journal.pgph.0002361)
Supplement: Attachment — Submitted filename: BRAVA PlosGPH RevResponse v2.docx [file pgph.0002361.s002.docx]

**Department of Medicine**

**Division of Infectious Diseases**

13 November 2023

Nirbhay Kumar PhD

Academic Editor, *PLOS Global Public Health*

RE: PGPH-D-23-01564

Dr Kumar:

This letter accompanies the re-submission of our manuscript entitled “Analytic optimization of *Plasmodium falciparum* marker gene haplotype recovery from amplicon deep sequencing of complex mixtures.” We appreciate the chance to revise the manuscript after your reviewers’ comments.

My co-authors Zena Lapp, Elizabeth Freedman, Kathie Huang, Christine Markwalter, Andrew Obala, and Wendy Prudhomme-O’Meara approved submission of this version of the manuscript.

Below we have enumerated our point-by-point responses to those reviewer comments. For each comment, the reviewer comments are italicized, and our responses are indented and in normal text, with specific text changes underlined. In addition, the tracked document includes other updates for clarity.

We’ve included tracked and clean versions of the revised text.

**Reviewer 1**

1. *The conclusion in the abstract, that haplotypes can be successfully recovered, seems at odds with the data presented showing a failure to recover 35% of the haplotypes in this controlled environment.*

RESPONSE: We have updated the abstract to read: “Taken together, our observations indicate that, with sufficient read depth, the majority of haplotypes can be successfully recovered from DBS while limiting the false positive rate.”

2. *The conceptual framework of this paper seems very narrow, calling haplotypes in this one species. I understand this focus and its public health relevance, but the paper misses an opportunity to relate this work to the broader concept of haplotype calling that is also key in other infectious diseases, e.g., HIV, HCV, COVID-19, etc. Furthermore, there are extensive simulation studies that offer insights into the generally poor performance of haplotype callers (e.g.,* [*https://doi.org/10.1016/j.meegid.2020.104277*](https://urldefense.com/v3/__https:/doi.org/10.1016/j.meegid.2020.104277__;!!OToaGQ!o5MFWLN8culXvgZUzTqrK_qSCIQBY6-F6YGEV9IOs62nZwDCwTWTJ0T0kQJ1RNnJXzAcR9DuVKZxOidfkayF$)*). The paper would be better served and more broadly applicable if it related the study findings to this broader context and more extensive literature on haplotype callers. If the authors prefer to keep the focus on this one species, that’s fine, but then I think this is not the right venue for this paper. For the broader readership of PLoS Public Health, it is important to provide linkages and insights beyond this one species. This is especially important this this sort of study has already been done in this species. The authors extend the insights by having a larger mock community and getting data from blood spots, but in the end, the conclusions are very similar.*

RESPONSE: We agree with the reviewer that more context related to other use cases for haplotype calling in global health is warranted; we added this information throughout the manuscript. However, we kept the focus of the manuscript on malaria parasites because we do not have evidence to claim that our results are directly generalizable to other pathogens. The changes are:

Abstract: “Molecular epidemiologic studies of malaria parasites and other pathogens commonly employ amplicon deep sequencing (AmpSeq) of marker genes derived from dried blood spots (DBS)”

Introduction: “Similarly, these methods are used in molecular epidemiologic studies of viral and bacterial pathogens.”

Discussion: “AmpSeq is an increasingly popular tool for molecular epidemiologic studies of various pathogens including *P. falciparum* collected on DBS”

Conclusion: “These observations can be used to guide analysis and interpretation both of *P falciparum* haplotypes recovered from DBS but also of other pathogens who share with malaria parasites high genetic variability, multiplicity of strains, and informative genetic markers.”

We would also like to emphasize that this manuscript did not intend to compare haplotype callers. We added this clarification to the Introduction: “With these reads, we employed an existing tool for haplotype inference to and investigated…” We have also changed the name of the GitHub repository to “haplotype_recovery_experiment” in case the previous name was confusing.

3. *Line 117, please provide BioProject number.*

RESPONSE: We have added the BioProject number (PRJNA1008913). The sequences will be available upon publication.

4. *Line 121, GitHub link does not work, thus, the pipeline is not available for review.*

RESPONSE: Thanks for checking out the GitHub link. We tested the link on several devices and it appears to work for us. We converted it to a hyperlink in the text in case that helps. If there are still issues with accessing the repository, please let us know.

5. *Line 128, the authors use P.f. 3D7 as the reference genome to which why map reads and call haplotypes. Other studies (not cited) have shown that the reference genome can make a big difference in the number of haplotypes called. The authors might explore this aspect. There are a large number of P.f. genomes available. The choice of 3D7 is not justified. Assuming the authors chose this genome for a good reason (it is geographically and/or temporally relevant to ongoing malaria outbreaks (?), they might do something like estimate a phylogeny of the P.f. genomes available on GenBank (~34) and then choose another that is evolutionarily distant from the 3D7 and rerun their pipeline to see the impact on haplotype calls. This should be a pretty straightforward analysis that is just a change in the reference genome and then re-running the established pipeline, doesn’t cost extra lab supplies, and would provide some additional insights with respect to dependence of inferences on the reference genome choice for haplotype calling.*

RESPONSE: We chose 3D7 because it is a commonly used reference sequence, is common in our field site in Western Kenya, and is included in the reference strains used in this study. We believe that the reference strain should not make a difference in haplotype calling in this study because we were able to recover the majority of expected haplotypes. However, the reviewer brings up a good point. We believe it is best practice to choose a reference sequence that is closely related to the sequences present in a given study. Therefore, for this study we selected as a second reference sequence the most distantly related sequence to 3D7 amongst the reference strains we used (HB3). 3D7 originated from West Africa and HB3 originated from Latin America. The genetic pairwise distance between these strains for the markers we used ranged from 4 (for *sera2*) to 10 (for *msp7* and *ama1*). When we called haplotypes using reads mapped to the HB3 reference sequence, we recovered exactly the same haplotypes as with 3D7. Additionally, in >99% of cases, the read depth was identical for both as well. We have added this to the manuscript:

Methods: “In a sensitivity analysis, the mapping was repeated using the HB3 reference genome.”

Results: “The haplotypes returned were identical when reads were mapped to either the 3D7 or to the HB3 reference genome.”

6. *Line 145, the systematic error definition seems to be measuring the error rate in the Sanger sequencing approach to estimating ‘truth’ in haplotypes. This is the fundamental flaw with these sorts of empirical studies compared to computer simulation, you don’t actually know the ‘truth’ and therefore true/false positive rates are really hard to get to. Rather than ignoring these data, I recommend the authors report these results as well as some direct comparison of Sanger versus DBS for identifying haplotypes. This makes the whole haplotype censoring (lines 158-177) statistics strange because you clearly don’t have reasonable estimates of ‘true positives’ if you have a bunch of true positives that you are throwing out of the analysis because you didn’t see them with Sanger sequencing. This whole like of logic seems flawed to me.*

RESPONSE: We agree with the reviewer that computer simulation is the gold standard in knowing the truth of what haplotypes are present in a sample; however, computer simulation does not capture many important aspects of stochasticity present in experimental and field data. We also agree that Sanger sequencing may have missed minority haplotypes in reference strains. We have added this as an explicit limitation in the Discussion: “First, it is possible that multiple haplotypes were present in the original template strains and were missed during Sanger sequencing, a limitation of this sequencing method.”

However, the haplotypes we classify as systematic error may not have been present in the original template strains, but may have been introduced at a later step - during gDNA extraction of the mixtures. This hypothesis is supported by the fact that many of the strains we define as systematic error were reference strains present in other mixtures. We have added this information to the Methods: “These were suspected to be truly present owing to either inadvertent introduction to mixtures during gDNA preparation or the presence of multiple haplotypes in the original source gDNA. In most cases, the sequence of these haplotypes was that of a reference strain that was not expected to be present in the mixture, supporting the former over the latter hypothesis.”

Furthermore, we cannot compare Sanger sequencing to deep sequencing because we performed Sanger sequencing only on the original template strain to determine the target reference sequences, while we performed deep sequencing only on the mixtures. We clarify this in the Methods: “The products of each individual strain were Sanger sequenced to determine the reference sequence for each strain” and “Where possible, the Sanger sequences were validated against publicly-available sequences. These sequences were defined as the true reference sequence for each strain, and this the reference strain haplotypes (n=5 per strain, 1 for each marker).”

Finally, we do not ignore the systematic haplotypes, but rather describe their presence in great detail (Figure 2) and only omit them when attempting to optimize censoring criteria. For that part, we do not feel comfortable claiming that haplotypes we do not know for sure were in the original mixture were “true positives”, but we also don’t want them to influence our assessment of reasonable censoring criteria as we believe they are not due to random error, which is what the censoring criteria are designed to filter out.

7. *Lines 179-188 – what is the ‘risk factor’ for Sanger sequencing relative to the haplotypes that looked real with the DBS?*

RESPONSE: We did not compare Sanger sequencing to deep sequencing. Instead, we used the individual sequences identified by Sanger sequencing to determine whether or not the sequence was identified in each of the deep-sequenced mixtures (see #6 above). We also add clarification to the Methods about the risk factor analysis:

“In order to identify what factors were associated with the failure to recover a haplotype from a mixture, we performed a bivariate and multivariate logistic regression of risk factors for haplotype missingness…”

“…risk factors were target, starting proportion of the reference template strain, read depth (per 10,000 reads), parasite density, and expected number of distinct haplotypes present in the sample.”

8. *Lines 216 – 219 are all repeat of the methods.*

RESPONSE: We have included a brief summary of methods to improve readability, with the assumption that most readers won’t delve deeply into the Methods sections.

9. *Lines 224 – 226 are method repeats as well.*

RESPONSE: See #8 above.

**Reviewer 2**

10. *The supplemental data is poorly annotated. The authors direct the reader to a website which contains some of the data, but the various files are not referenced at all within the text and it difficult to try to sort out what they did or what the files mean. The lack of callouts to supplemental tables is nonstandard and makes review difficult. For example, what exactly is dada2_long.csv? I can sort of figure it out, but it is not really the reviewers’ responsibility to spend hours deconvoluting the authors work and to try to interpret what is contained in poorly labeled tables. It really is responsibility of the corresponding author to make sure this is done. The various intermediate files on the website should be given table numbers and there should be explicit callouts to these table numbers throughout the text. For example, the authors write “The products of each were Sanger sequenced to determine the reference sequence for each strain and the results of the sequencing are given in Table SY.” These data showed that our results were similar (or identical?) to sequences deposited in PlasmoDB for X of the strains, correctly calling each of the X expected variants. In another example, the authors write in the legend to Figure 1, “Pairwise single nucleotide variant (SNV) distances between reference haplotypes of each of the marker genes obtained by Sanger sequencing” but then fail to mention that this raw data appears to be contained in a file called “ref_pairwise_dists.csv” on their github site. Pairwise distance ref is also a confusing name. What they actually show is the number of SNVs distinguishing two clones/strains across all queried markers.*

RESPONSE: Apologies for sowing confusion. The GitHub link is not supplementary material in the standard sense of providing additional supporting information. Instead, it is provided for reproducibility to enable readers to recreate the analyses and figures present in the manuscript. This is why we do not reference the files and folders contained there anywhere in the manuscript. We have clarified this in the Methods: “Code and data to recreate the analyses and figures in this manuscript can be found at….” In addition, we have updated the [README](https://github.com/duke-malaria-collaboratory/haplotype_recovery_experiment) on the GitHub repo to make it clearer exactly what is contained in the corresponding files and folders, and added a [README](https://github.com/duke-malaria-collaboratory/haplotype_recovery_experiment/tree/main/data) to the data directory describing exactly what is in each of the files and what figures and tables they were used to generate.

11. *The authors should not duplicate material that is in the main text either. I see a file called “target overview” which seems to be an exact clone of Table 2. It is work for the reviewer to go through the tables and putting duplicated tables into the supplement wastes the reviewers’ time. The figures are also duplicated on the website.*

RESPONSE: See #10 above.

12. *Overall. I am a bit confused about how the authors define a haplotype. For a given strain such as FCR3, given that this is a haploid organism, there should be only 1 haplotype, right? So if you properly sequence 10 haploid clonal strains, you should get exactly 10 reference haplotypes, even if you sequence five different marker genes for each. The authors mention that they have 34 reference haplotypes. Does this mean there were new alleles that were not detected by Sanger sequencing? Or are they creating extra artificial haplotypes by linking FCR3 ama1 to Dd2 csp? It would be easier to understand if the authors mentioned that there were X variable sites in the dataset with a total of Y different queried SNVs for the 10 strains. Overall, the focus on haplotypes instead of called variants makes it this more difficult to understand. I assume 34 is a subset of the possible 50, reduced by the fact that some strains (FCR3 and FCB) appear to be identical to one another in this analysis? Is this expected?*

RESPONSE: We have chosen to focus on haplotypes rather than SNVs as this is a common way of using AmpSeq for molecular epidemiologic studies of *P. falciparum*. We have clarified this in the Introduction: “Malaria parasite surveillance and molecular epidemiologic studies increasingly employ as a genotyping approach amplicon deep sequencing (AmpSeq) of short polymorphic fragments of parasite DNA to identify haplotypes present in a sample.”

The reviewer is correct that there is one haplotype per marker per strain, which means that there are 50 total haplotypes, including 37 (not 34) distinct haplotypes as some strains contained the same identical haplotype for a given marker. We have clarified this in the Methods:

“For each reference strain and marker (n = 50), ….. These sequences were considered to be the true target reference sequences for each strain, and thus the reference strain haplotypes (n = 5 per strain, 1 for each marker).”

“We define a haplotype as a unique sequence returned by DADA2 (as described in the previous section).”

“Expected haplotype: A haplotype with an identical sequence to that of a template sequence (reference haplotype) expected to be observed in the sequenced library."

13. *The authors should potentially make sure input DNA quality was the same for all samples and comment on this. Did some samples have higher random error rates?*

RESPONSE: We did not perform further internal QA, and as now described below in #17, we have clarified that input DNA was directly from the vendor following Quality Assurance.

14. *I would like to test to see if there were more errors in repetitive, recombinogenic regions of each marker gene sequence or if errors were distributed across the length of the amplicon*.

RESPONSE: We compared the mutations we observed to mutations found in Pf6k genomes and observed that point mutations of false positive haplotypes were relatively evenly distributed across the sequence. In the plot below, the gray lines are variability from Pf6k and the red lines are the start and end of the sequence.

15. *Please indicate what REF means in Table 3 legend.*

RESPONSE: We have added as a footnote in Table 3: “REF: reference group for each comparison.”

16. *Table 2 is insufficient to allow readers to duplicate the authors work because nucleotide positions can change in different PlasmoDB version. Typically, the PlasmoDB version is given and the database cited as well.*

RESPONSE: We have added the PlasmoDB version to the table footer and cited PlasmoDB: “* Coordinates correspond to those from PlasmoDB [REF]”

17. *The authors do not indicate what “MRA-1169G” is. I can determine that it is the BEI resource catalog number but they should say so.*

RESPONSE: Indeed, they are from BEI Resources, and we have amended the Methods to read: “… from each of ten reference P. falciparum strain genomic DNAs (gDNAs), each obtained from BEI Resources and accompanied by Certificates of Analysis: MRA-102G (3D7)…”

18. *Table 1. There seems to be no explanation for how the authors arrived at the number of Pf6k variant positions which can be influenced by a variety of parameters. Please include parameters in table legend. Please reference Pf6K as well in the Table.*

RESPONSE: We have updated Methods to be clearer exactly how we counted variants: “From Pf6k (REF) VCF files, we extracted and tallied the variant positions that passed filtering (i.e. FILTER=PASS) in the amplified portion of each marker gene.” We have also amended the Table 2 legend to read: “** Includes all variants that passed filtering in the amplified region”

19. *The author’s sequence data does not seem to have been deposited in an appropriate sequencing repository*.

RESPONSE: See #3 above.

20. *It would also be good if the authors did a bit of QC on their sequencing results ahead of time to make sure they were sequencing the right things. How well did their unmixed sequencing match the sequences for the strains in PlasmoDB? It is, afterall, common for strains to get mixed up. Were the parasites cloned before DNA was isolated for sequencing?*

RESPONSE: We used genomic DNA acquired directly from BEI resources, each of which is accompanied by a Certificate of Analysis to authenticate identity, typically by amplification and sequencing of a polymorphic locus like *msp2*. We have added this as in #17 above.

21. *I tried to check some of their data and ended up confused. EP2-110 has two reads listed in their dada2_long.csv but their metadata file indicates that four different strains went into the sequencing but there is an MOI of 3, so it is not clear what is actually shown in dada2_long.csv. There are other cases where the MOI is supposed to be 4 and there are five sequences listed. I am confused. Both EB2-107 reads are listed as “random error.” Is this the only sequence that obtained for ama1 and 3D7? Have things been removed? Again, a detailed description of the file, and how it was created would be necessarily to understand what happened.*

RESPONSE: See #10 above.

We are much obliged for your (re)consideration of our manuscript.

Warm regards,

Steve M Taylor MD MPH

Associate Professor of Medicine and Global Health

Duke University
