# Supplementary material for: Analytic optimization of Plasmodium falciparum marker gene haplotype recovery from amplicon deep sequencing of complex mixtures
Source: PLOS Glob Public Health. 2024 May 30;4(5):e0002361. doi: 10.1371/journal.pgph.0002361 (PMC11139333; doi:10.1371/journal.pgph.0002361)
Supplement: Attachment — Submitted filename: BRAVA PlosGPH Rev2Response v1.docx [file pgph.0002361.s003.docx]

**Department of Medicine**

**Division of Infectious Diseases**

7 March 2024

Nirbhay Kumar PhD

Academic Editor, *PLOS Global Public Health*

RE: PGPH-D-23-01564

Dr Kumar:

This letter accompanies the re-submission of our manuscript entitled “Analytic optimization of *Plasmodium falciparum* marker gene haplotype recovery from amplicon deep sequencing of complex mixtures.” My co-authors Zena Lapp, Elizabeth Freedman, Kathie Huang, Christine Markwalter, Andrew Obala, and Wendy Prudhomme-O’Meara approved submission of this version of the manuscript.

The only reviewer comment was: *The authors have done a fine job responding to my suggestions from the first review. The GitHub repo is now accessible. However, the BioProject is not. That will need to be fixed before publication. Otherwise, they've done a nice job of tightening up the manuscript.*

RESPONSE: The BioProject is public, and it is find-able on NCBI as PRJNA1008913.

Because the manuscript has not changed we have not submitted a tracked manuscript document. Everything is the same as before. We are much obliged for your (re)consideration of our manuscript.

Warm regards,

Steve M Taylor MD MPH

Associate Professor of Medicine and Global Health

Duke University
